# Cutaneous Metastasis As the Initial Clue to Lung Adenocarcinoma: A Case Report

**DOI:** 10.7759/cureus.115117

**Published:** 2026-08-24

**Authors:** Sowmya Sridhar, Divya Raviprakash, Priya Dharshini R, Leena Joseph, Sudha Rangarajan

**Affiliations:** 1 Department of Dermatology, Sri Ramachandra Institute of Higher Education and Research, Chennai, IND; 2 Department of Pathology, Sri Ramachandra Institute of Higher Education and Research, Chennai, IND

**Keywords:** cutaneous metastasis, histopathology, immunohistochemistry, lung adenocarcinoma, positron emission tomography

## Abstract

Cutaneous metastasis is an uncommon but clinically significant manifestation of internal malignancy, often indicating advanced disease and poor prognosis. Its varied clinical morphology often mimics benign dermatological conditions, posing a diagnostic difficulty. In certain cases, cutaneous metastasis can be the sentinel sign of an undetected primary tumor. We report the case of a 65-year-old woman with a prior history of treated cervical carcinoma who presented with a new-onset skin lesion over her shoulder. Subsequent histopathological, immunohistochemical, and radiological evaluations confirmed cutaneous metastasis arising from a new primary lung adenocarcinoma rather than recurrence of the cervical carcinoma. This case highlights the importance of meticulous histopathological and immunohistochemical evaluation in patients with a history of malignancy presenting with a new, atypical cutaneous lesion. Even after a prolonged disease-free interval, such lesions may represent secondary metastasis from a distinct primary malignancy rather than recurrence of the original tumor. Early recognition of cutaneous metastasis provides an important diagnostic clue to an underlying primary malignancy and facilitates timely management.

## Introduction

Cutaneous metastasis is a rare clinical presentation with an incidence ranging from 0.7% to 10% [1]. In patients with internal malignancy, cutaneous metastasis usually develops months to years after diagnosis of the primary tumor, with the most common primary tumors being breast cancer, melanoma, and lung cancer [2]. In some cases, cutaneous metastasis may be the first symptom before the primary malignancy is diagnosed. Clinically, cutaneous metastases do not have a uniform characteristic appearance, making it difficult to diagnose. Histopathological examination with appropriate immunohistochemical evaluation is therefore essential for establishing the diagnosis and identifying the primary tumor. We present a rare case of cutaneous metastasis from lung adenocarcinoma in a patient with previously treated cervical carcinoma, highlighting the importance of considering a new primary malignancy when evaluating cutaneous nodules.

## Case presentation

A 65-year-old woman presented to the dermatology outpatient department with complaints of painful, raised skin lesions over the right shoulder and scalp for four months. The pain was persistent and not relieved by over-the-counter nonsteroidal anti-inflammatory drugs. She also reported unintentional weight loss and decreased appetite for the past one year. The patient had a history of stage IIIB cervical carcinoma, for which she had undergone total abdominal hysterectomy with bilateral salpingo-oophorectomy followed by 30 fractions of radiotherapy in 2012. She had remained asymptomatic for 13 years following completion of treatment.

**Figure 1:**
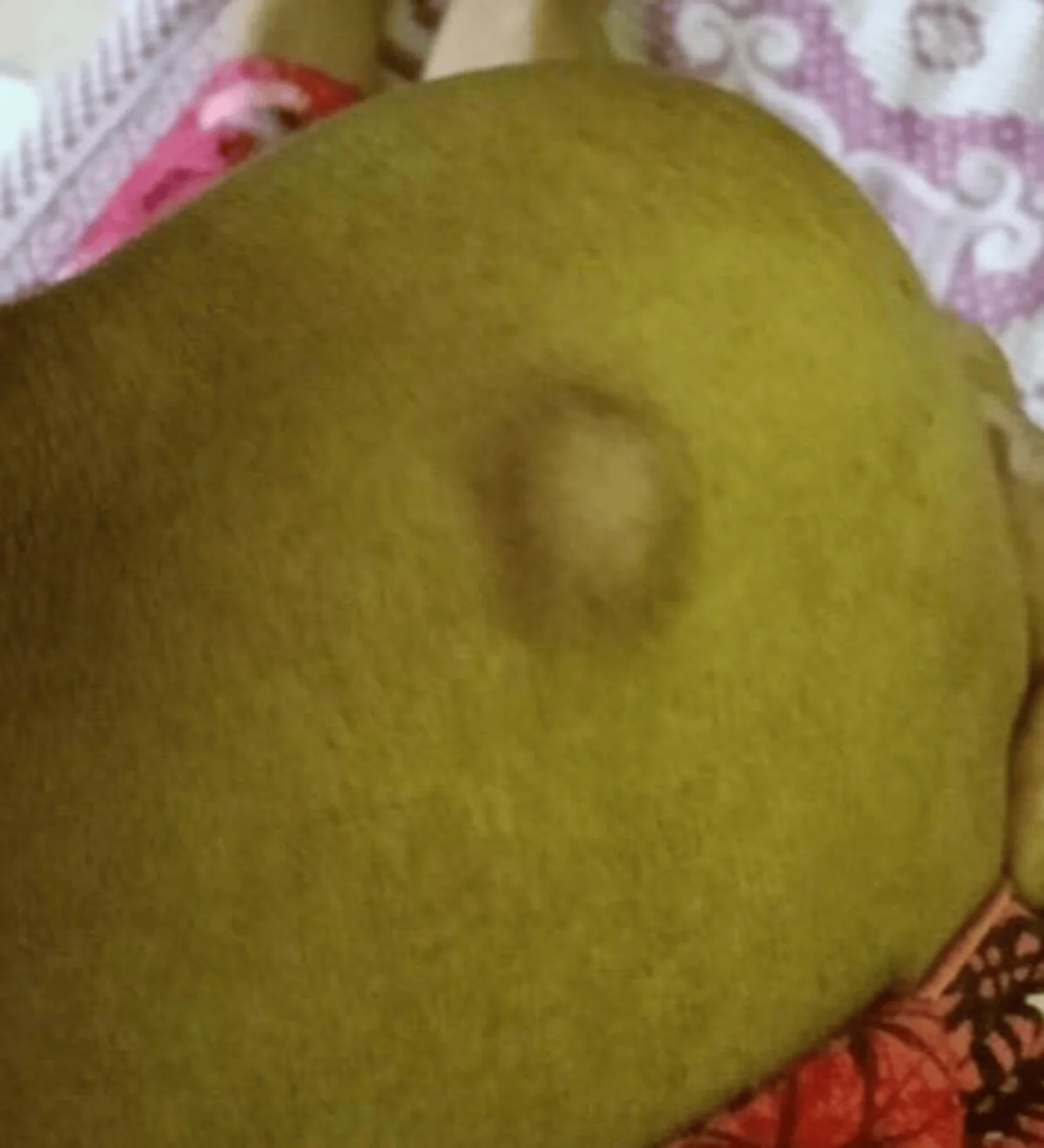
Solitary nodule over the right shoulder

Cutaneous examination revealed a single erythematous, shiny, hard, immobile nodule measuring approximately 1 × 1 cm over the right shoulder (Figure 1).

Additionally, multiple boggy, ill-defined hard swellings were noted over the frontal and left temporal scalp. The skin overlying the lesions was not pinchable. No regional lymphadenopathy was noted.

**Figure 2:**
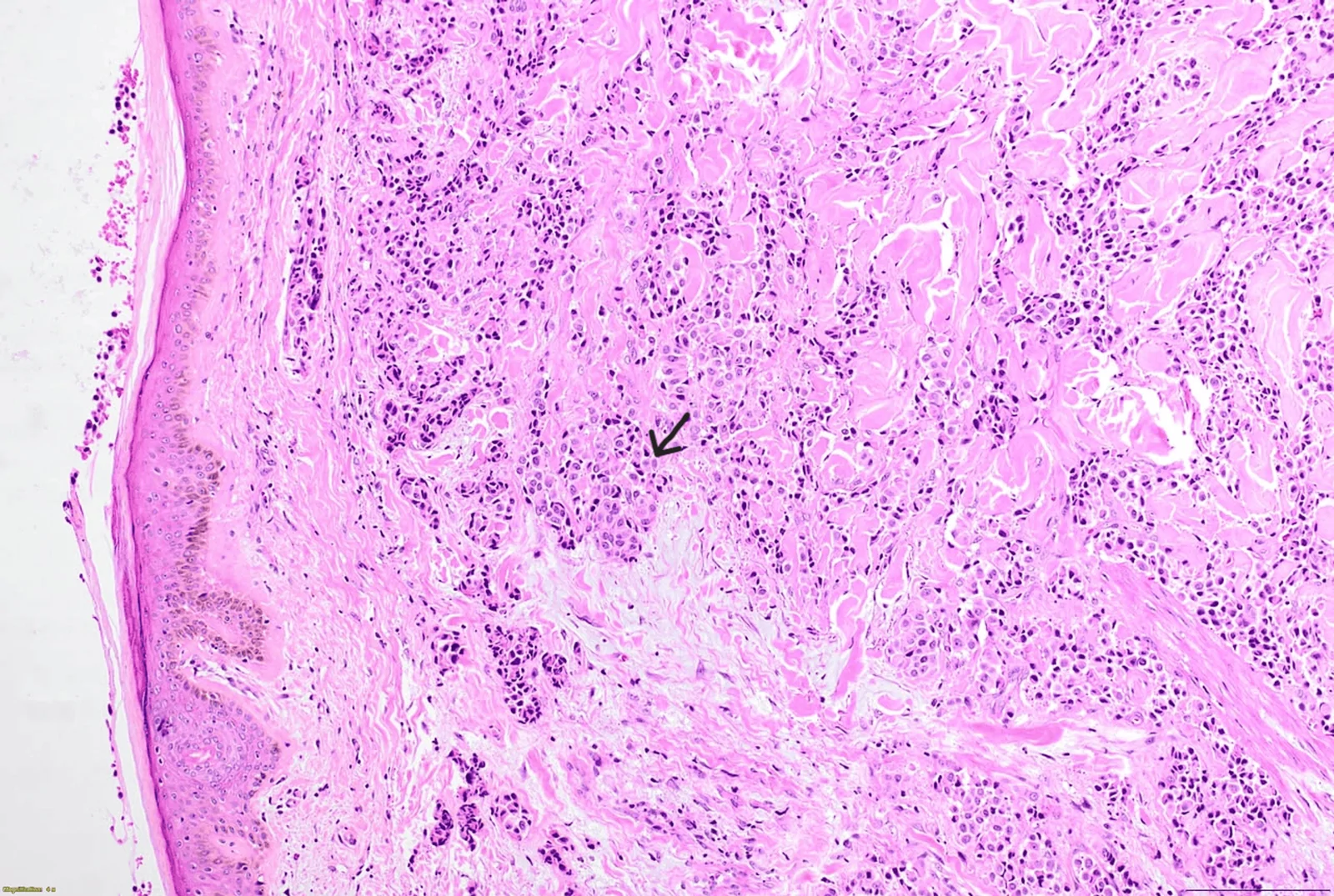
Histopathological examination (hematoxylin and eosin staining, ×100) showing the epidermis with underlying nests and sheets of tumor cells between collagen bundles (black arrow)

**Figure 3:**
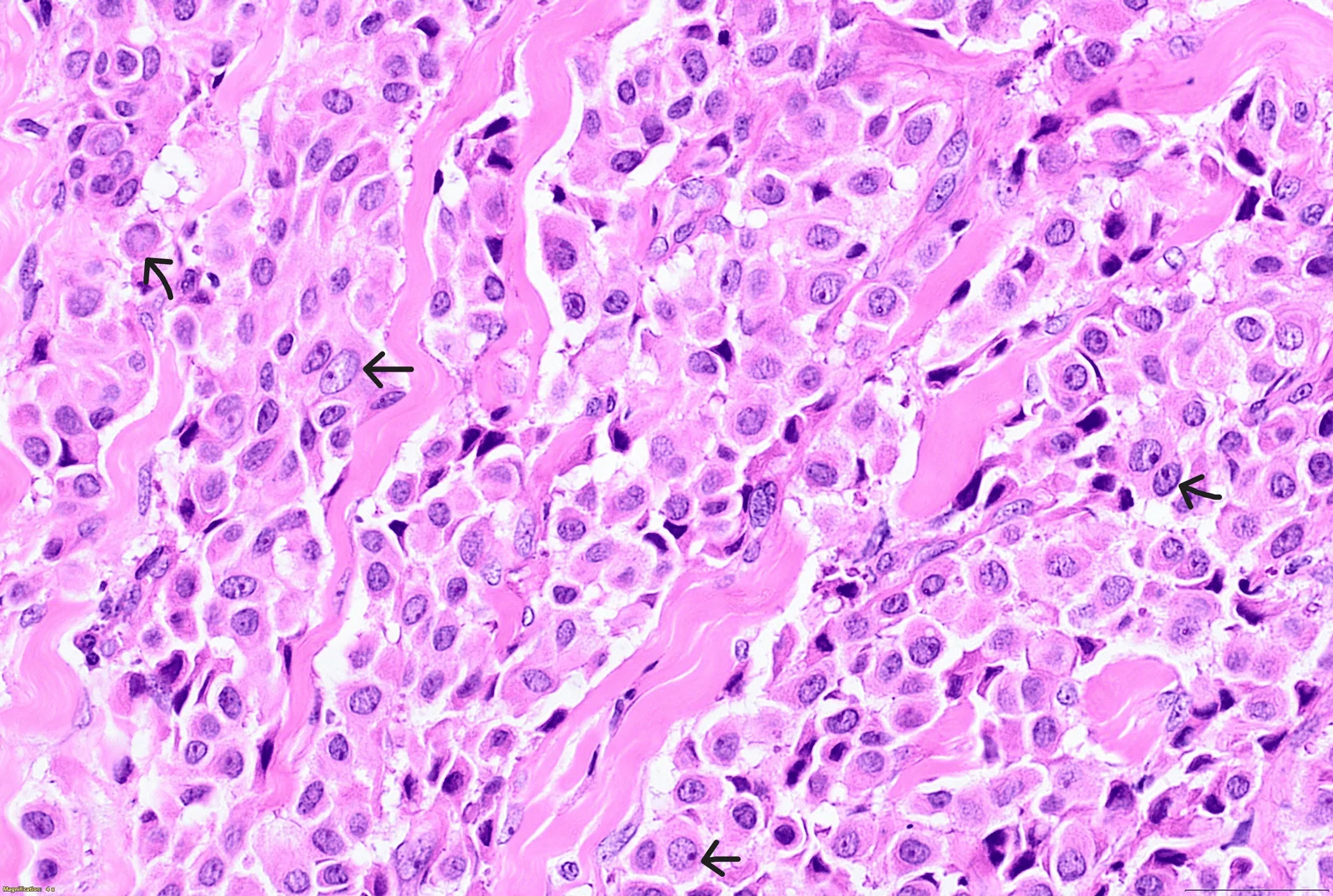
Histopathological examination (hematoxylin and eosin, ×400) showing tumor cells with nuclear pleomorphism (black arrows) at higher magnification

Given the past history of cervical carcinoma and the clinical suspicion of cutaneous metastasis, a gynecological evaluation was undertaken to rule out recurrence of squamous cell carcinoma. Gynecological examination did not reveal any signs of local tumor recurrence, and a whole-body PET-CT scan was advised to confirm the diagnosis. A 4-mm punch biopsy was obtained from the nodular lesion over the right shoulder. Histopathological examination revealed infiltration of the subepithelial tissue by malignant cells arranged in sheets and nests, consistent with metastatic adenocarcinoma. The tumor cells showed moderate pleomorphism with an increased nuclear-to-cytoplasmic ratio (Figures 2-3).

**Figure 4:**
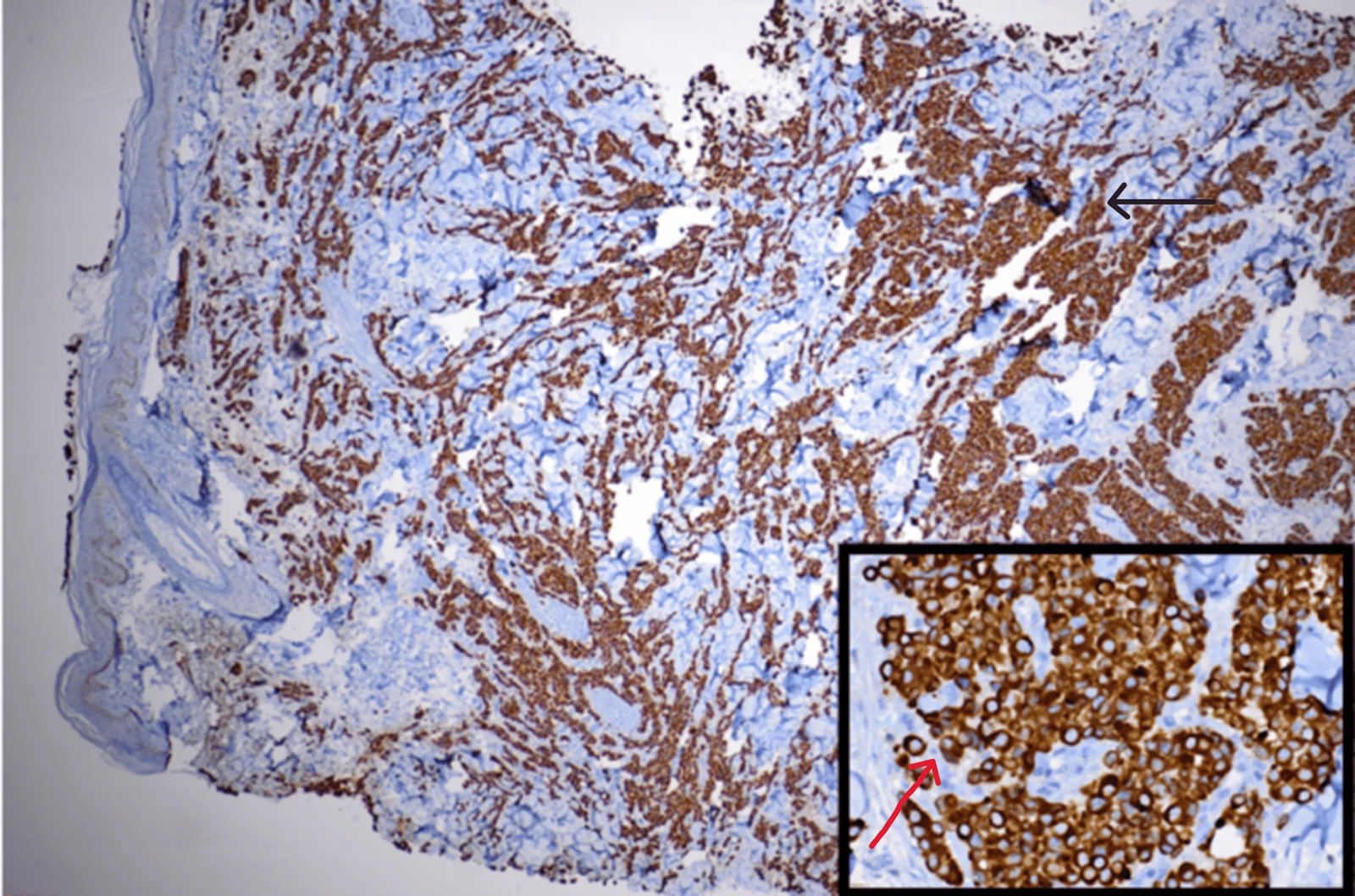
Immunohistochemical staining showing CK7 positivity in tumor cells (black arrow) at ×40, with a higher magnification inset highlighting the staining pattern (red arrow) at ×400

**Figure 5:**
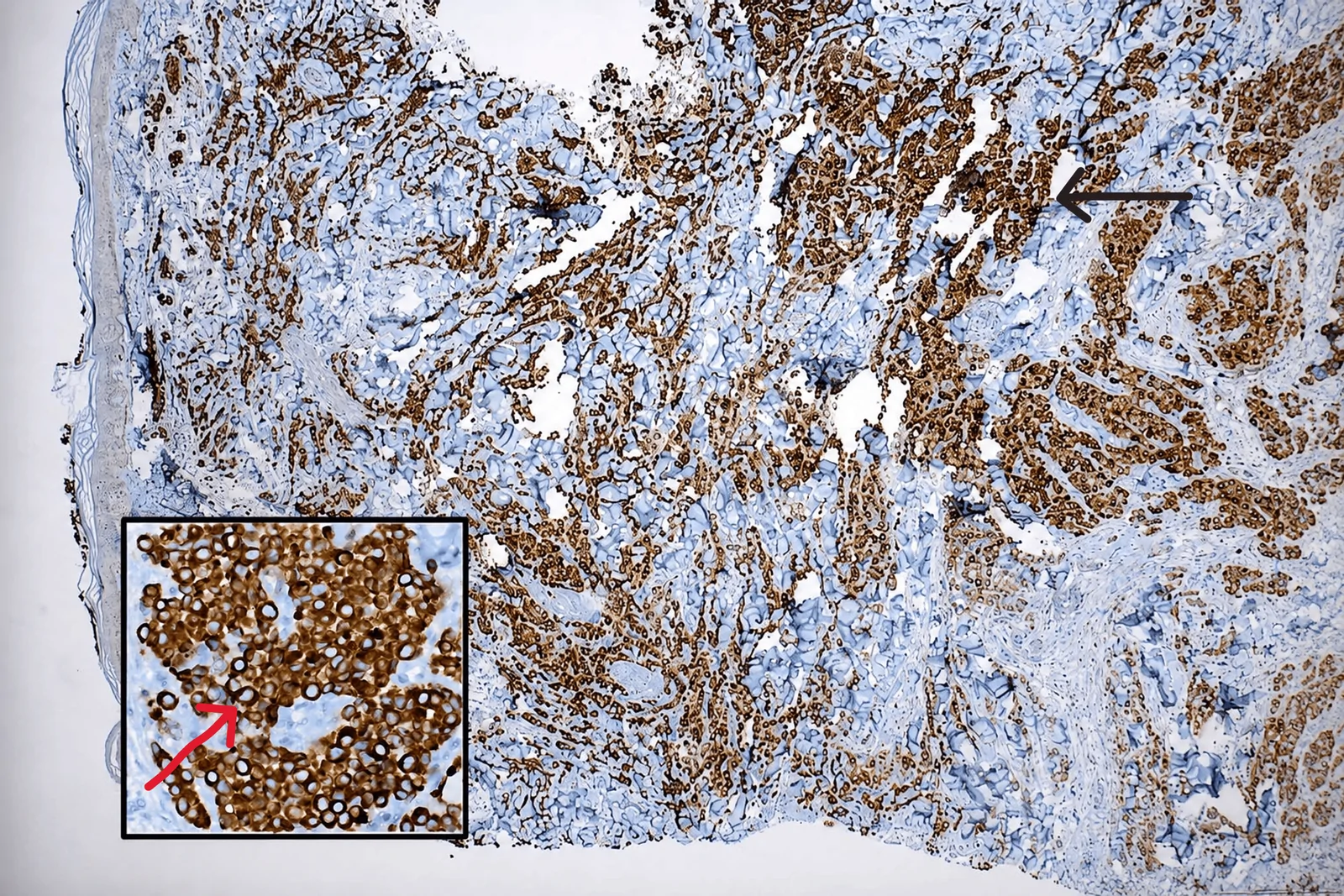
Immunohistochemical staining showing CK20 positivity in tumor cells (black arrow) at ×40, with a higher magnification inset highlighting the staining pattern (red arrow) at ×400

In view of the poorly differentiated malignancy, immunohistochemical staining for CK20, CK7, SOX-10, p40, and Ki-67 was performed; staining was positive for CK7 and CK20 (Figures 4-5).

The histopathological and immunohistochemical findings favored a diagnosis of cutaneous metastasis, with a possible primary lung adenocarcinoma. Whole-body PET-CT findings were suggestive of a primary lung malignancy with metastatic deposits in lymph nodes, liver, bone, and skin. Following the PET-CT, the patient was initiated on supportive palliative care in view of advanced malignancy. Despite intervention, the disease progressed, and the patient succumbed to the illness.

## Discussion

**Figure 6:**
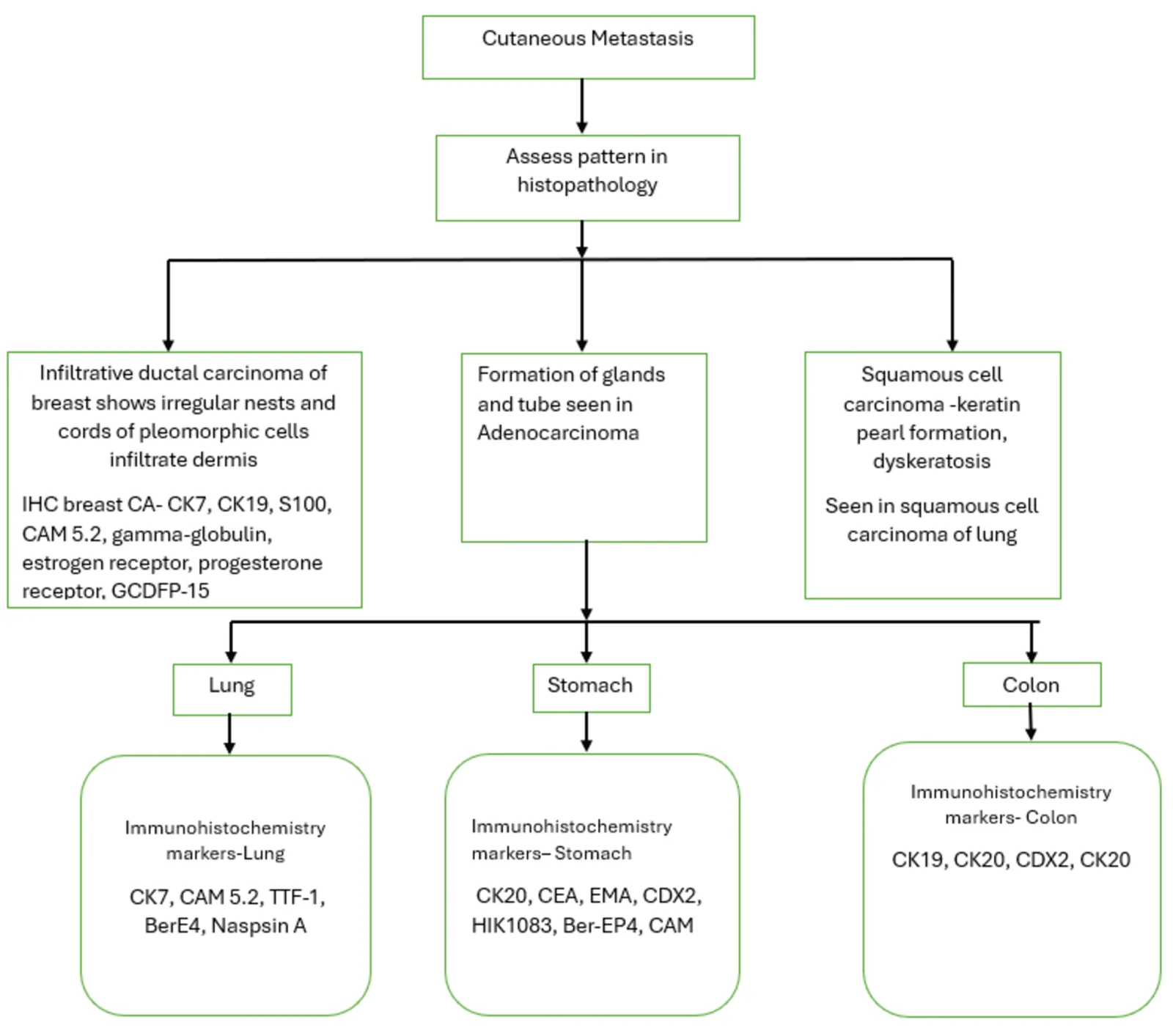
Diagnostic approach to cutaneous metastasis based on histopathological and immunohistochemistry findings IHC: immunohistochemistry, CK: cytokeratin, CA: carcinoma, S100: S100 calcium binding protein, CAM 5.2: cytokeratin CAM 5.2, CDX2: caudal-type homeobox 2, Ber-EP4: epithelial cell adhesion molecule antibody, CEA: carcinoembryonic antigen, EMA: epithelial membrane antigen, GCDFP-15: gross cystic disease fluid protein 15, HIK1083: gastric mucin monoclonal antibody HIK1083, TTF-1: thyroid transcription factor 1 [6-8]

Cutaneous metastasis from internal malignancies is an uncommon clinical phenomenon, with a reported incidence ranging from 0.7% to 10% [1]. Its presence is often a harbinger of advanced systemic disease and carries a poor prognosis. The primary sources of skin involvement vary significantly by gender. In women, breast cancer, colorectal cancer, and melanoma are the most frequent precursors. In men, the most common sources include lung cancer, melanoma, and colorectal cancer [2]. Cutaneous metastasis occurs through lymphatic or hematogenous dissemination of malignant cells and usually signifies an advanced stage of neoplastic disease. Clinically, lesions most commonly present as painless, firm, solitary or multiple nodules; however, their morphology is highly heterogeneous and may mimic benign or inflammatory dermatoses, including ulcerative lesions, morphea-like sclerotic plaques, erysipelas-like indurated erythema (carcinoma erysipeloides), alopecia neoplastica, or telangiectatic tumor nodules and embolic acral lesions [3]. Given the rich vascular supply of the scalp, head and neck, and upper extremities, these sites are frequent locations for metastatic deposits [4]. A systematic diagnostic approach is essential, particularly when cutaneous lesions represent the first manifestation of malignancy. A detailed medical history should include prior radiation exposure, carcinogen exposure, and previous malignancies and should be accompanied by a thorough cutaneous examination. Dermoscopy may reveal polymorphous vascular patterns such as linear irregular, dotted, or arborizing vessels, along with white streaks, globules, or pigmented structures, aiding differentiation from primary adnexal tumors [5]. Laboratory evaluation may include a complete blood count, serum lactate dehydrogenase, calcium levels, and relevant tumor markers (e.g., carcinoembryonic antigen, prostate-specific antigen, and alpha-fetoprotein). However, histopathological examination remains the gold standard for diagnosis. Microscopically, cutaneous metastases characteristically demonstrate dermal infiltration by pleomorphic malignant cells with frequent mitoses and relative sparing of the epidermis. Immunohistochemistry of formalin-fixed, paraffin-embedded tissue shows characteristic marker profiles [6]. The diagnostic algorithm is summarized in Figure 6.

Management is primarily palliative and multidisciplinary, focusing on treatment of the underlying malignancy. Cutaneous lesions often regress with systemic therapy, including chemotherapy, targeted agents (e.g., epidermal growth factor receptor inhibitors, anaplastic lymphoma kinase inhibitors, and human epidermal growth factor receptor 2 inhibitors), and immunotherapy (e.g., checkpoint inhibitors), in selected cancers. Topical imiquimod and 5-fluorouracil have also been used on solitary lesions. Other local modalities such as palliative radiotherapy, electrochemotherapy, or surgical excision may be considered for symptomatic or solitary lesions. Despite intervention, the presence of cutaneous metastasis portends a poor prognosis, with a median survival ranging from three to twelve months [9-11]. In our case, cutaneous metastasis was the initial manifestation of lung adenocarcinoma, with the patient presenting with a nodular lesion over the shoulder. While undergoing diagnostic evaluation and receiving supportive palliative care for symptomatic control, the patient experienced rapid clinical decline and succumbed to the illness before definitive anticancer treatment could be initiated. Patients with a history of treated internal malignancy, as in our patient, require long-term follow-up and regular dermatological screening to facilitate prompt diagnostic evaluation of suspicious cutaneous lesions. Cutaneous metastasis generally indicates advanced disease and is associated with a poor prognosis, although outcomes vary according to the primary tumor, systemic involvement, and response to therapy. This case highlights that cutaneous metastases can occasionally precede local symptoms of the primary malignancy. Despite the generally poor prognosis associated with metastatic disease, this finding underscores the importance of early recognition and prompt evaluation of atypical skin lesions, as they may serve as the first clue to an underlying internal malignancy.

## Conclusions

Histopathological examination with targeted immunohistochemical evaluation established the diagnosis of cutaneous metastasis from lung adenocarcinoma, leading to the identification of the underlying primary malignancy. The case highlights the importance of correlating cutaneous findings with histopathology and immunohistochemistry when a skin lesion presents as the initial manifestation of an internal malignancy. Early recognition of such cutaneous manifestations can facilitate timely oncological referral and appropriate systemic management.
